# The DSF type quorum sensing signalling system RpfF/R regulates diverse phenotypes in the opportunistic pathogen *Cronobacter*

**DOI:** 10.1038/srep18753

**Published:** 2016-01-04

**Authors:** Angela Suppiger, Athmanya Konegadde Eshwar, Roger Stephan, Volkhard Kaever, Leo Eberl, Angelika Lehner

**Affiliations:** 1 Department of Microbiology, University of Zurich, CH-8008 Zurich, Switzerland; 2 Institute for Food Safety and Hygiene, University of Zurich, CH-8057 Zurich, Switzerland; 3 Research Core UnitMetabolomics, Hannover Medical School, D-30625 Hannover, Germany

## Abstract

Several bacterial pathogens produce diffusible signal factor (DSF)-type quorum sensing (QS) signals to control biofilm formation and virulence. Previous work showed that in *Burkholderia cenocepacia* the RpfF_Bc_/RpfR system is involved in sensing and responding to DSF signals and that this signal/sensor gene pair is highly conserved in several bacterial species including *Cronobacter* spp. Here we show that *C. turicensis* LMG 23827^T^ possesses a functional RpfF/R system that is involved in the regulation of various phenotypes, including colony morphology, biofilm formation and swarming motility. *In vivo* experiments using the zebrafish embryo model revealed a role of this regulatory system in virulence of this opportunistic pathogen. We provide evidence that the RpfF/R system modulates the intracellular c-di-GMP level of the organism, an effect that may underpin the alteration in phenotype and thus the regulated phenotypes may be a consequence thereof. This first report on an RpfF/R-type QS system of an organism outside the genus *Burkholderia* revealed that both the underlying molecular mechanisms as well as the regulated functions show a high degree of conservation.

Members of the genus *Cronobacter* spp. are considered opportunistic pathogens associated with rare but severe neonatal systemic infections predominantly in pre-term and/or low birth weight infants and thus have attracted the attention of public health authorities and researchers in the past^1^,^2^,^3^.

Epidemiological investigation of outbreaks of *Cronobacter* spp. infections in hospitals indicated powdered infant formula as a source of contamination, when these organisms were isolated from both reconstituted milk as well as from milk feeding equipment and utensils. The latter may be enhanced by the organism’s ability to adhere and form biofilms on many surfaces, including silicone, latex, polycarbonate (used in the feeding bottle manufacture) and stainless steel^4^,^5^.

Bacterial processes involved in biofilm formation and virulence, are often controled by quorum sensing (QS), a mechanism based on the production, release and detection of signaling molecules of low molar mass. Extracellular concentrations of signal molecules are sensed by the bacteria and, upon reaching a population density-dependent threshold, they are detected by the cells, which in turn induce target gene expression in a coordinated fashion^6^,^7^,^8^,^9^.

To date many structurally unrelated signal molecules have been identified, including *N*-acyl-homoserine lactones (AHLs) in Gram-negative bacteria, oligopeptides in many Gram-positive bacteria and autoinducer-2 (AI-2), which is thought to serve as a signal for interspecies communication^7^,^8^,^9^.

Another group of signal molecules are the *cis*-2-unsaturated fatty acids, often referred to as DSF (diffusible signal factor) family signals^10^. The first fatty acid signal, *cis*-11-methyl-2-dodecenoic acid, was identified in the culture supernatant of the phytopathogen *Xanthomonas campestris* pv. *campestris* (*Xcc*)^11^. Subsequently, fatty acid-based QS-systems were also identified and in members of the genera *Xylella* and *Stenotrophomonas*^12^,^13^ where they were shown to control the production of virulence factors^11^. More recent work showed that *Burkholderia cenocepacia* produces the signal molecule *cis*-2-dodecenoic acid, which was named BDSF (*Burkholderia* diffusible signal factor)^14^. BDSF is synthesized by the enoyl-CoA hydratase RpfF_Bc_^15^ and is sensed by the receptor protein RpfR, which contains PAS-GGDEF-EAL domains^16^. Binding of BDSF to the PAS domain stimulates the c-di-GMP phosphodiesterase activity of RpfR, which in turn lowers the intracellular c-di GMP level. This signal transduction relay is very different from the one originally described for *X. campestris*, in which the DSF receptor RpfC is a hybrid sensor kinase that phosphorylates its cognate response regulator RpfG. This regulator contains in addition to a REC domain a HD-GYP domain, which is responsible for the c-di-GMP phosphodiesterase activity of the protein^17^.

Interestingly, homologs of RpfF_Bc_ and RpfR are present not only in many *Burkholderia* species but also in strains belonging to the genera *Achromobacter*, *Yersinia*, *Serratia*, *Enterobacter* and *Cronobacter*^16^, suggesting that RpfF/R type signaling systems may by far more widespread than anticipated. In this study we analysed the RpfF/R system of the clinical strain *Cronobacter turicensis* LMG 23827^T^, and show that it is involved in the regulation of biofilm formation, macrocolony morphology, proteolytic activity and virulence.

## Results and Discussion

### RpfF directs the synthesis of a DSF family signal molecule and negatively regulates intracellular c-di-GMP levels in C. turicensis

**Figure 1:**
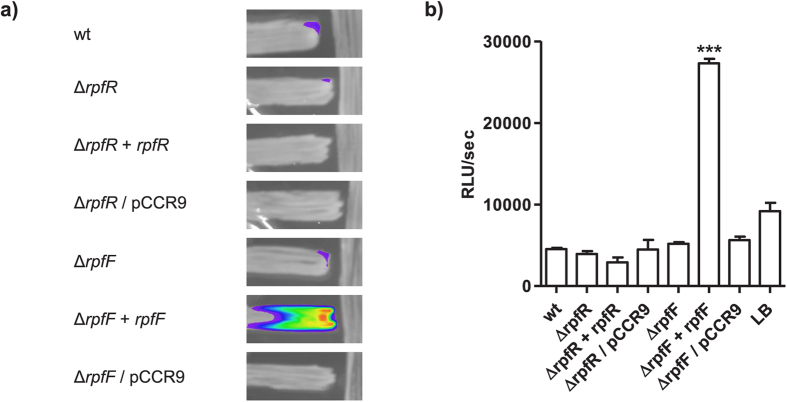
Overexpression of *rpfF* activates the biosensor *B. cenocepacia* H111 –rpfF_Bc_/pAN-L15, which is capable of detecting various DSF family signals. (**a**) The *C. turicensis* LMG 23827^T^ wild type (wt), the mutants (Δ*rpfF,* Δ*rpfR),* the complemented mutants (Δ*rpfF* + *rpfF,* Δ*rpfR* + *rpfR*) and the mutants carrying the empty vector (Δ*rpfF*/pCCR9, Δ*rpfR*/pCCR9) (vertical) were tested in cross-streak experiments against the biosensor (horizontal). The biosensor was clearly induced by the complemented *rpfF* mutant (Δ*rpfF* + *rpfF)*. (**b**) The strains were also tested for the production of DSF family molecules in liquid assays. As with the cross streaking, induction of the biosensor was only observed with the Δ*rpfF* + *rpfF* strain. Error bars indicate SEM, n = 4; *P < 0.05 (ANOVA, oneway).

Previous work identified homologs of both RpfR and RpfF from *B. cenocepacia* in *C. turicensis* LMG 23827^T16^. To investigate the role of this putative QS system in this organism we constructed defined mutants as well as genetically complemented derivatives thereof. We tested the strains for the production of DSF family signal molecules by the aid of the *Burkholderia*-based biosensor H111–rpfF_Bc_ pAN-L15 both in cross-streaking and liquid culture experiments. Under the conditions tested the wild type strain did not induce the biosensor. However, the complemented *rpfF* mutant, in which the wild type allele is expressed from a plasmid, clearly induced the biosensor (Fig. 1), suggesting that RpfF directs the biosynthesis of a *cis*-2 fatty acid signal molecule. We hypothesize that under standard laboratory conditions the amount of signal released by the wild type strain is below the detection limit of our bioassay but that the complemented strain, in which *rpfF* is expressed from a plasmid, produces sufficiently high amounts to induce the biosensor.

**Figure 2:**
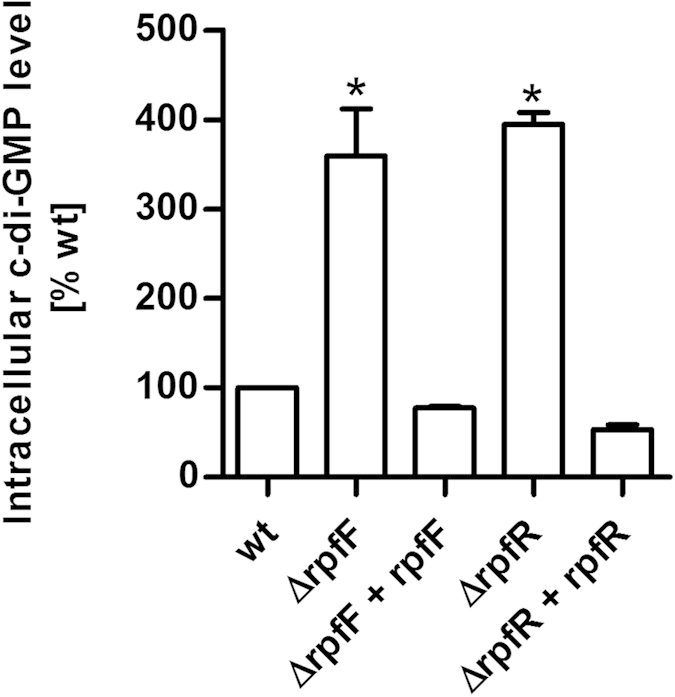
RpfR and RpfF affect the intracellular c-di-GMP level. The intracellular c-di-GMP level was significantly increased in the *rpfR* and *rpfF* mutants relative to the wild type. Detection was performed by by LC-MS/MS. Error bars indicate SEM, n = 2; *P < 0.05 (ANOVA, oneway).

RpfR family proteins contain a GGDEF as well as an EAL domain which are associated with the synthesis and degradation of c-di-GMP respectively^18^. The *C. turicensis* LMG 23827^T^ RpfR homolog CBA31265 exhibits an identical domain structure. In order to evaluate the role of RpfR in this strain the intracellular c-di-GMP levels were determined in the wild type, the *rpfR* and *rpfF* mutants and in the complemented strains Δ*rpfR* + *rpfR* and Δ*rpfF* + *rpfF*. The intracellular c-di-GMP level of the *rpfR* and *rpfF* mutants was found to be 3.9-fold or 3.4-fold increased relative to the wild type. Genetic complementation of the mutant reduced the c-di-GMP level to the level of the wild type. These results suggest that both RpfR and RpfF have a negative effect on the intracellular c-di-GMP level (Fig. 2). This is in agreement with the finding that RpfR in *B. cenocepacia* exhibits a net phosphodiesterase activity^16^.

### RpfF/R plays a role in quorum sensing regulated phenotypes in C. turicensis LMG 23827^T^

**Figure 3:**
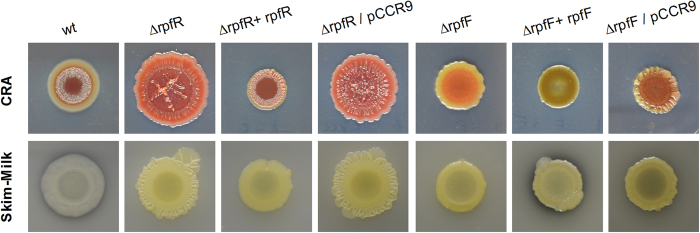
The RpfF/R QS-system controls colony morphology and protease production. Deletion of *rpfR* induced a rough, wrinkly colony morphology and increased EPS production on Congo red agar plates (CRA, upper panel). Both the Δ*rpfR* and the Δ*rpfF* mutant showed reduced protease production on skim-milk plates compared to the wild type (lower panel).

**Figure 4:**
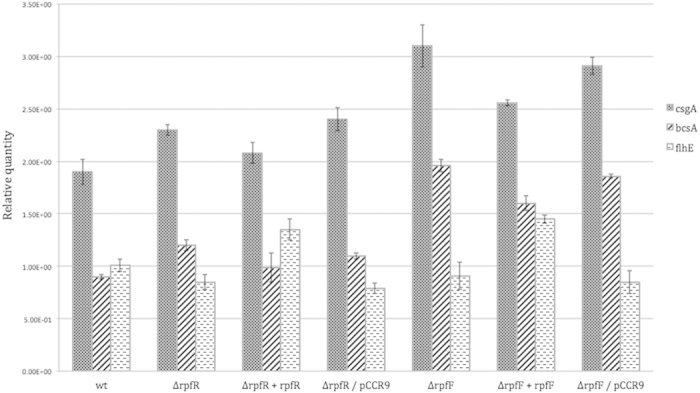
RT-qPCR analysis of *csgA, bcsA* and *flhE* gene expression in *C. turicensis* LMG 23827^T^ (wt), the mutants (Δ*rpfF,* Δ*rpfR),* the complemented mutants (Δ*rpfF* + *rpfF,* Δ*rpfR* + *rpfR*) and the mutants carrying the empty vector (Δ*rpfF/*pCCR9, Δ*rpfR/*pCCR9). The respective mRNA levels were normalized to the 16S rRNA reference gene. Error bars indicate SEM, n = 3.

We next investigated whether the RpfF/R system is involved in the regulation of typically QS-associated phenotypes. In contrast to the wild type we observed a rough colony morphology of the *rpfR* and to a lesser degree with the *rpfF* mutant on Congo red agar plates (Fig. 3). The strong pinkish colour of the *rpfR* mutant relative to the wild type may suggest an increased production of cellulose and/or curli^19^. In order to support this hypothesis we performed expression studies targeting the gene coding for the catalytic subunit of the cellulose synthase *bscA* as well as the major curli subunit *csgA*. Expression of both genes was considerably increased in the mutants compared to the wild type. However, complementation only partially restored their expression (Fig. 4).

**Figure 5:**
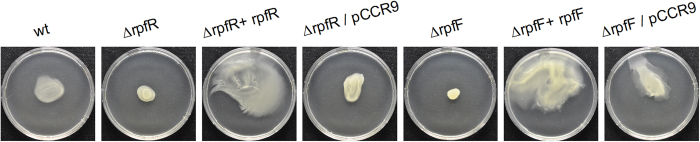
Overexpression of the RpfF/R QS-system increased swarming motility. Strains were spot inoculated on 0.4% NYG agar and plates were photographed after 24 h incubation.

In addition, we observed that both mutants showed reduced proteolytic activity (Fig. 3). Swarming motility on NYG +0.4% agar was not significantly affected by inactivation of the RpfF/R system. However, the complemented mutants exhibited increased swarming motility (Fig. 5). These results are supported by the results of the RT qPCR experiments targeting the flagellar regulon-associated gene *flhE*, which was unaltered in the *rpfF/R* mutants but significantly higher in the complemented mutants (Fig. 4). This finding may be explained by a dose effect due to the additional copies of this gene in the complemented mutants.

**Figure 6:**
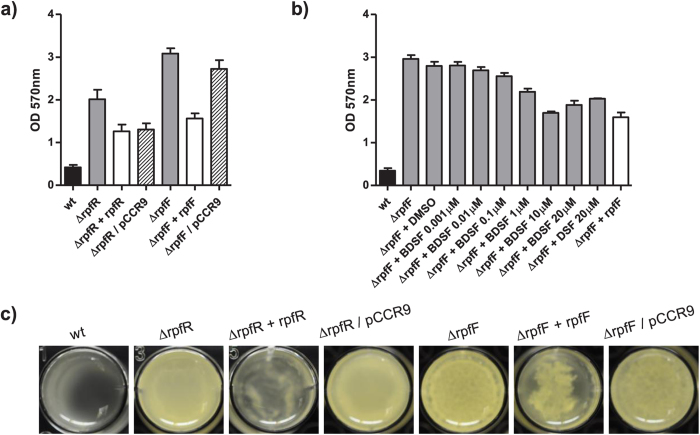
The RpfF/R system regulates biofilm formation under static conditions. (**a**) Deletion of either *rpfR* or *rpfF* increased biofilm formation in microtiter plates. (**b**) Partial restoration was obtained by genetic complementation or by supplementing the medium with BDSF (0.001 μM–20 μM) or DSF (20 μM). Error bars indicate SEM, n > 2. (**c**) Both RpfR and RpfF are involved in pellicle formation tested in NYG broth at room temperature for 48 h.

Both mutants formed significantly more biofilm under static conditions in microtiter plates (Fig. 6a) than the parental strain. Complementation of the mutants partially restored the wild type phenotype. In the study by Hartmann *et al.* (2010)^20^ genes involved in biofilm formation in the closely related species *Cronobacter sakazakii* were identified using a transposon mutagenesis approach. *BscA* and *flhE* were – amongst others – two of the genes that were found to contribute to biofilm formation. Our expression analysis performed in this study suggests that *bcsA* but not *flhE* is regulated by the rpfF/R system (Fig. 4).

Importantly, the strains Δ*rpfR* + *rpfR* and Δ*rpfF*/pCCR9 showed growth defects and did not reach the same OD as the other strains, which may explain the poor complementation of the Δ*rpfR* mutant. The growth curves of wild type strains, complemented mutants and mutants carrying the pCCR9 vector are depicted in Supplementary Figure S1. Partial restoration was also observed when the *rpfF* mutant was supplemented with at least 1 μM BDSF or 20 μM DSF (Fig. 6b). We also tested the various strains for pellicle formation, i.e. biofilm formation at the liquid-air interface. Both mutants showed increased pellicle formation and complementation restored the wild type behavior (Fig. 6c). In a study by Lehner *et al.* (2005)^21^ it has been reported that cellulose is one of the major components present in pellicles formed in *Cronobacter* spp. strains. The increased expression levels of *bcsA* in the mutants as observed in our study suggest a negative influence of the RpfF/R regulon in *C. turicensis* biofilm formation. This is in contrast to the homologous system of *B. cenocepacia*^16^ but similar to the genetically different DSF-dependent RpfCG systems of *Stenotrophomonas maltophilia* E77 or *X. campestris* pv. *campestris*^22^,^23^.

### Zebrafish infection studies

**Figure 7:**
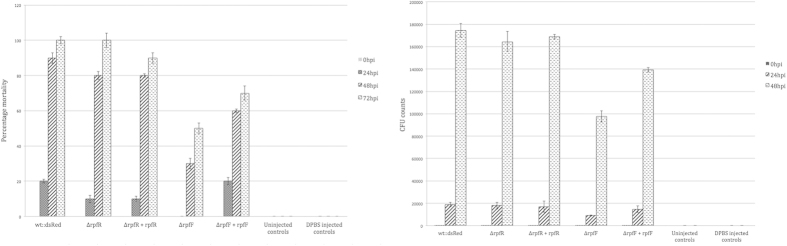
(**a**) Survival rates of zebrafish larvae injected with 50 CFU *C. turicensis* LMG 23827^T^/pRZT3::dsRed (wt::dsRed), the mutants Δ*rpfF* and Δ*rpfR* as well as the the complemented mutants Δ*rpfF* + *rpfF* and Δ*rpfR* + *rpfR*. A set of DPBS injected as well uninjected embryos served as controls. Error bars indicate SEM, n = 3. (**b**) Mean growth curve of *C. turicensis* LMG 23827^T^/pRZT3::dsRED (wt::dsRed), the mutants Δ*rpfF* and Δ*rpfR* as well as the complemented mutants (Δ*rpfF* + *rpfF,* Δ*rpfR* + *rpfR)* inside infected zebrafish larvae with a starting inoculum of approx. 50 CFU. A set of DPBS injected as well uninjected embryos served as controls. Error bars indicate SEM, n = 3

We tested the Δ*rpfF* and the Δ*rpfR* mutants for pathogenicity in a zebrafish infection model. The dsRed-labeled wild type strain *C. turicensis* LMG23827^T^ (wt::dsRed) served as control. The mortality rate of the zebrafish larvae at 48 hpi increased to approximately 90% for injection with wt::dsRed whereas the mortality rate decreased to 50% when the larvae were infected with the mutant Δ*rpfF* (Fig. 7a). Furthermore, the bacterial load was significantly lower with the *rpfF* mutant when compared with the wild type control (Fig. 7b) indicating a role of the RpfF/R system in the expression of virulence factors required for pathogenicity in the zebrafish model. Injection experiments using the complemented mutant strain (Δ*rpfF* + *rpfF*) resulted in higher mortality rate and higher bacterial load, whereas control experiments using the mutant strain transformed with the vector alone (Δ*rpfF*/pCCR9) yielded mortality rates and bacterial loads comparable to the ones observed in the Δ*rpfF* mutant experiments (data not shown).

Intriguingly, the mortality rate of larvae injected with the Δ*rpfR* strain was virtually indistinguishable from the wild type.

Here we have shown that *C. turicensis* posesses a RpfF/R family QS system which relies on a *cis*-2-unsaturated fatty acid signal molecule. RpfF/R-type QS systems are particularly widespread among members of the genus *Burkholderia*^10^. We analysed for the first time a RpfF/R family QS system in a bacterium not belonging to the genus *Burkholderia* and demonstrated that despite the phylogenetic distance (β *versus* γ subdivision of proteobacteria) both the molecular mechanism as well as the regulated phenotypes are very similar. Like in *B. cenocepacia*, the RpfF/R system was found to affect swarming motility, biofilm formation and virulence in *C. turicensis*. Furthermore, in both organisms the QS system modulates the intracellular secondary messenger c-di-GMP and this in turn appears to regulate the observed QS-dependent phenotypic traits. The finding that the *rpfF* but not the *rpfR* mutant reduced the virulence of *C. turicensis* suggests that an alternative signal receptor may be present in this strain. This is not unprecedented, as in *B. cenocepacia* an alternative BDSF receptor, BCAM0227, has been identified that is used by some strains as a parallel signaling system to control a subset of functions^24^. However, a bioinformatic analysis neither identified a homolog of BCAM0227 nor of *rpfC*, the DSF receptor of *Xcc*^25^.

In conclusion, our data provide evidence that RpfF/R-type QS systems are not restricted to *Burkholderia* sp. but may be widespread among Gram-negative bacteria, in which they influence surface colonization and virulence through modulation of the intracellular c-di-GMP levels. It will be of interest to investigate if homologous systems in other bacteria will control the same phenotypes.

## Material and Methods

### Bacterial strains and culture conditions

*C. turicensis* LMG 23827^T^ 26, a clinical isolate responsible for two fatal sepsis cases in neonates in Zurich in 2006 was used in the study. Strains *C. turicensis* LMG 23827^T^_Nal^R^ as well as *C. turicensis* LMG 23827^T^/pRZT3::dsRed were described previously^27^,^28^.

For selection purposes, during zebrafish embryo infection experiments, *C. turicensis* LMG 23827^T^_Δ*rpfF*/pCCR9 as well as *C. turicensis* LMG 23827^T^_Δ*rpfR*/pCCR9 were constructed by transformation of the strains with the vector using standard methods.

Strains were grown in Luria–Bertani (LB) broth over night at 37 °C with gentle shaking. Where appropriate, culture medium or agar was supplemented with nalidixic acid at 256 mg L^−1^ (*C. turicensis* LMG 23827^T^_Nal^R^), chloramphenicol at 30 mg L^−1^ (strains harbouring pDS132) or both (transconjugant strains) or tetracyclin at 50 mg L^−1^ (strains harbouring pCCR9 or pRZT3::dsRed).

For microinjection experiments, the bacteria were harvested by centrifugation at 5000 × *g* for 10 min and washed once in 10 ml of Dubelcco’s phosphate buffered saline (DPBS, Life Technologies, Switzerland.) After a second centrifugation step, the cells were resuspended in DPBS, and appropriate dilutions were prepared in DPBS.

### DNA extraction and manipulations

Chromosomal DNA was isolated using the DNeasy Blood and Tissue kit, plasmids were extracted with the QIAprep Spin Miniprep or Plasmid Midi kits following the manufacturer’s instructions. For purification purposes (PCR, restriction digest, agarose gel purification) the Qiagen MinElute PCR Cleanup kit or MinElute Gel Purification kit was employed. Enzymes and respective buffers were obtained from Roche Molecula Diagnostics (Rotkreuz, Switzerland) and used according to the manufacturer’s instructions.

### Construction of C. turicensis LMG 23827^T^ in frame deletion mutants

Bacterial strains, plasmids and primers used for the construction of mutants are listed in Supplemental Table S1. Deletion mutants of *C. turicensis* LMG 23827^T^ *rpfF* (CTU_23310) and *rpfR* (CTU_23300) genes were constructed following the protocol described by Philippe *et al.* (2004)^29^. Details are provided in the supplementary material.

### Phenotypic assays

Colony morphology: Overnight cultures grown in LB were adjusted to an OD_600_ = 1.0 in AB minimal medium^30^. 5 μl of this cell suspension was spottet on CRA plates (2 g Casamino acids, 0.3 g yeast extract, 80 μl of 1 M MgSO_4_, 4 g agar, dH_2_O ad 200 ml, supplemented with 1.6 ml congo red (0.5% in 50% EtOH), 0.65 ml coomassie blue (0.3% in 50% EtOH). The plates were incubated at room temperature for six days before colonies were photographed.

Protease production: Cells of an overnight culture were resuspended in LB and 5 μl cell suspension was spottet on skim milk plates (1% LB agar, 2% w/v skim milk powder). Plates were incubated at 37 °C for two nights and then kept at room temperature.

Swarming motility: Analysis was performed as previously described by Deng *et al.* (2012)^16^, except motility was monitored on NYG plates containing 0.5% peptone, 0.3% yeast extract, 2% glycerol and 0.4% agar.

Biofilm formation: Overnight cultures were washed and diluted to an OD_600_ = 0.01 in AB minimal medium supplemented with 0.4% glucose and 0.5% casamino acids^30^. 100 μl samples were added to 96 well plates incubated statically for 18 h at 30 °C. Growth was measured at 550 nm in a plate reader (Synergy HT; Bio-Tek, Germany). Surface attached cells were stained by the addition of 100 μl of 1% crystal violet for 30 min at room temperature. The plate was washed thoroughly with tap water and air-dried. To solubilize the stain, 120 μl DMSO was added to each well, incubated for 20 min at room temperature and OD at 570 nm was measured. Data are based on at least 2 independent experiments with 7 technical replicates each.

Bioassays for the production of *cis*-2 fatty acids by using the biosensor *B. cenocepacia* H111 –rpfF_Bc_/pAN-L15. This sensor is sensitive to nM levels of synthetic BDSF and is suitable to detect a wide range of *cis*-2 fatty acid molecules (Suppiger *et al.* submitted). In cross-streaking experiments both the test- and the sensor strain were streaked on LB agar plates close to each other to form a T. The plates were incubated overnight at 37 °C. Following the addition of 10 μl decanal to the lid of the plate the bioluminescence of the sensor strain was visualized using the NightOWL LB 983 (Berthold Technologies, Zug, Switzerland). In liquid bioassays the biosensor was grown in LB broth containing kanamycin 100 μg ml^−1^ to an OD_600_ of 2.0. Overnight cultures of the strains to be tested were centrifuged at 6000 rpm, 5 min and the supernatant (SN) was centrifuged again. 100 μl of this cell-free SN was mixed with 100 μl sensor and incubated for 20 hours at 30 °C. Relative luminescence units (RLU) were obtained by adding 1–2 μl Decanal (Sigma Aldrich, Buchs, Switzerland) to each well and detection was performed using a plate reader (Synergy HT; Bio-Tek, Germany).

### Intracellular cyclic-di-GMP level

Bacterial overnight cultures were subcultured in LB medium and 5 ml were harvested at an OD_600_ = 2.0 by centrifugation at 5000 rpm, 4 °C. Nucleotide extraction was performed as described by Spangler *et al.* (2010)^31^ with slight modifications: cXMP was omitted and the solvent was evaporated in Speedvac at 60 °C. Quantification was performed by LC-MS/MS^32^.

### Expression analysis of selected genes by RT-qPCR

The expression levels of the 16S rRNA, *csgA, bcsA,* and *flhE* genes in *Cronobacter turicensis* LMG23827^T^ wild type and its respective *rpfR* and *rpfF* mutants that were grown in AB medium supplementd with 0.4% glucose and 0.5% casamino acids at 30 °C to early stationary phase were determined using reverse transcription quantitative-PCR (RT-qPCR). 1.5 ml of the above bacterial suspension was re-suspended in 0.5 ml of the lysis buffer of the RNeasyPlus Mini Kit (Qiagen, Hilden, Germany). The samples were transferred on to the lysing bead matrix in MagNA lyser tubes and mechanically disrupted (1 min at 6500 rpm) using the MagNA Lyser Instrument (Roche Molecular Diagnostics, Rotkreuz, Switzerland). RNA was isolated from the bacterial lysates following the RNeasy^Plus^Mini Kit protocol (Qiagen). Genomic DNA was removed by using a genomic DNA binding column and carrying out an on column DNAse I digestion. RNA was eluted in 50 μl of RNAse-free water, and subsequently quantified and quality controlled using the Nanodrop and BioAnalyzer instruments, respectively. 100 ng of RNA were reverse transcribed to cDNA using the Quantitect Reverse Transcription Kit (Qiagen). Residual DNA contamination was ruled out in each RNA sample by including a control in which the RT enzyme was omitted. Quantitative PCR was performed on 2.5 ng cDNA using the SYBR green I kit (Roche Molecular Diagnostics), and primers that are listed in Supplemental Table S2 in the LC480 (Roche Molecular Diagnostics) instrument. Following RT PCR conditions were applied for all four genes: 5 min 95 °C followed by 40 cycles of 95 °C for 10 seconds, 50 °C for 20 seconds, 72 °C for 20 seconds, 78 °C for 1 second. Quantification was performed using the Light Cycler 480 Relative Quantification Software (Roche Molecular Diagnostics). The *csgA, bcsA, flhE* mRNA levels were normalized using 16S rRNA as reference gene^27^.

### Zebrafish infection studies

Zebrafish (*Danio rerio*) strains used in this study were albino lines. Husbandry, breeding and microinjection of approx. 50 CFU of bacteria into the yolk sac of 2 dpf embryos was performed following the procedure described in the study by Fehr *et al.* (2015)^28^.

A set of uninjected embryos, incubated in E3 maintenance medium (5 mM NaCl, 0.17 mM KCl, 0.33 mM CaCl_2_, 0.33 mM MgSO_4_) was included in order to determine the quality of the embryos; embryos injected with DPBS served as controls. Injected embryos were transferred into 24-well plates (1 embryo per well) in 1 ml E3 medium per well, incubated at 28 °C and observed for signs of disease and survival under a Leica M165 C stereomicroscope twice a day. In order to follow the course of infection embryos or larvae were collected at several time points, namely at 0, 24, 48 and 72 h post infection (hpi) and individually treated for bacterial enumeration.

Research was conducted with approval (NO 216/2012) from the Veterinary Office, Public Health Department, Canton of Zurich (Switzerland). The applied methods were carried out following the approved guidelines.

### Bacterial enumeration by plate counting

The larvae were transferred to 1.5 ml centrifuge tubes and disintegrated by repeated pipetting and vortexing for 3 min in 1 mL of DPBS supplemented with 1% Triton X- 100 (Sigma-Aldrich, Buchs, Switzerland). Subsequently, serial dilutions of this mixture were plated onto LB plates supplemented with tetracycline 50 mg L^−1^ (strains harboring pCCR9 or pRZT3::dsRed). The plates were incubated up to 48 h at 37 °C.

### Survival assay

Embryos were microinjected as mentioned above and maintained individually in 24-well plates in E3 medium at 28 °C. The number of dead larvae was determined at different time points visually based on the absence of a heartbeat.

### Statistical analysis

Statistics and graphs were performed using GraphPad Prism 6 (GraphPad Software, San Diego, USA). Experiments were executed at least three times, unless stated otherwise. The CFU counts of individual larva at different time points and under different conditions were verified for significant variances by one-way ANOVA with Bonferroni’s post-test.

## Additional Information

**How to cite this article**: Suppiger, A. *et al.* The DSF type quorum sensing signalling system RpfF/R regulates diverse phenotypes in the opportunistic pathogen *Cronobacter*. *Sci. Rep.* **6**, 18753; doi: 10.1038/srep18753 (2016).

## Supplementary Material

Supplementary Information
