# Supplementary material for: The DSF type quorum sensing signalling system RpfF/R regulates diverse phenotypes in the opportunistic pathogen Cronobacter
Source: Sci Rep. 2016 Jan 4;6:18753. doi: 10.1038/srep18753 (PMC4698668; doi:10.1038/srep18753)
Supplement: Supplementary Information [file srep18753-s1.pdf]

Supporting information

The DSF type quorum sensing signalling system RpfF/R regulates diverse phenotypes in the opportunistic pathogen *Cronobacter*

Angela Suppiger[1], Athmanya Konegadde Eshwar[2], Roger Stephan[2], Volkhard Kaever[3], Leo Eberl[1*], Angelika Lehner[2*]

[1]Department of Microbiology, University of Zurich, CH-8008 Zurich, Switzerland

[2]Institute for Food Safety and Hygiene, University of Zurich, CH-8057 Zurich, Switzerland

[3]Research Core Unit Metabolomics, Hannover Medical School, D-30625 Hannover, Germany

* Corresponding authors email: lehnera@fsafety.uzh.ch , leo.eberl@botinst.uzh.ch

A. Suppiger and A. K. Eshwar contributed equally to the first authorship.

Construction of *C. turicensis* LMG 23827^T in frame deletion mutants

Primers were designed based on the whole genome sequence of *Cronobacter turicensis* LMG 23827^T (RefSeq accession numbers NC_013282 to NC_013285, GenBank accession numbers FN543093 to FN543096). Briefly, two flanking fragments (upstream, downstream) of the *rpfF* and the *rpfR* genes were amplified by PCR using oligonucleotide primers rpfFmut1modf (containing a *XbaI* recognition site), rpfFmut2r (containing a *XhoI* restriction site), rpfFmut3f (containing a *XhoI* recognition site), rpfFmut5r (containing a *XbaI* recognition site), and rpfRmut1f (containing a *XbaI* recognition site), rpfRmut2r (containing a *XhoI* recognition site), rpfRmut3f (containing a *XhoI* recognition site), and rpfRmut5r (containing a *XbaI* recognition site), respectively. The amplification mixes contained 0.4 mM of primers, 1 x AccuPrime (Invitrogen) buffer 2 (60 mM Tris-SO_4 (pH 8.9), 18 mM $(NH_4)_2SO_4$, 2 mM $MgSO_4$, 2 mM dGTP, 0.2 mM dATP, 0.2 mM dTTP, 0.2 mM dCTP, thermostable AccuPrimeTM protein, 1 % glycerol), 4 % dimethylsulfoxid (DMSO), 2 U AccuPrime Taq DNA Polymerase High Fidelity (Invitrogen) and 50 ng of template DNA. Following PCR conditions were used for the amplification: 95 °C for 120 s followed by 34 cycles of 95 °C for 30 s, 68 °C for 210 s and a final elongation step at 68 °C for 300 s. The resulting fragments were digested with *XbaI* and *XhoI* and ligated into the suicide vector pDS132 digested with *XbaI*. The constructs pDS132::Δ*rpfF* and pDS132::Δ*rpfR* were transformed into *E. coli* SM10 λpir via electroporation. The resulting strains *E. coli* SM10 λpir / pDS132::Δ*rpfF* or *E. coli* SM10 λpir_pDS132::Δ*rpfR* served as donor strains for conjugative transfer of the plasmid into *C. turicensis* LMG 23827^T_Nal^R. Transconjugants were selected on LB agar plates supplemented with both nalidixic acid 256 mg L^{-1} and chloramphenicol 30 mg L^{-1}. The genetic structure of the mutants was confirmed by the presence of two amplification products - one representing the chromosomal wild type *rpfF* or *rpfR* allele and a second product representing the truncated (Δ*rpfF,* Δ*rpfR*) allele originating from the (integrated) pDS132::Δ*rpfF* or pDS132::Δ*rpfR* vector - after PCR using primer pair rpfFContf, rpfFConr,

and rpfRConf, rpfRConr respectively employing the above mentioned AccuPrime amplification mixture (without DMSO) and following amplification conditions: 95 °C for 120 s followed by 32 cycles of 95 °C for 30 s, 54 °C for 210 s and a final elongation step at 68 °C for 300 s. The resulting amplification products were 825 bp (wt *rpfF* allel) and 110 bp (Δ*rpfF* allel) and 2185 bp (wt *rpfR* allel) and 217 bp (Δ*rpfF* allel) respectively.

Outcrossing was performed by plating serial dilutions of confirmed transconjugants onto LB agar plates supplemented with 5 % sucrose and no NaCl. Successful allelic exchange was verified in selected chloramphenicol sensitive and and sucrose resistant strains by the presence of the mutant allele after PCR using the above mentioned procedure.

For constitutive expression (complementation) of *rpfF* and *rpfR* in the respective mutants, the genes were amplified with primers rpfFComplf, rpfFComplr or rpfRComplf, rpfRComplr using the above mentioned Accuprime mixture (without DMSO) and following conditions: 95 °C for 120 s followed by 32 cycles of 95 °C for 30 s, 58 °C for 210 s and a final elongation step at 68 °C for 300 s.

The amplicons were digested with *BamHI* and *HindIII* (for *rpfF* cloning) or *BamHI* and *XbaI* (for *rpfR* cloning) and ligated into low copy vector pCCR9 digested with the respective enzymes.

Supplemental Table S1

Strains/plasmids/primers	Genotype/characteristic(s)/sequences	Source or reference
Mutant construction<br>Strains		
C. turicensis LMG 23827^T_Nal^R	Acceptor for transconjugation, Nal^R	Eshwar et al., 2015
E. coli SM10 λpir	Host for pDS132::Δ*rpfF*, pDS132::Δ*rpfR* construct generation; *thi, thr, leu, tonA lacY supE recA*::RP4-2-Tc::Mu, Km, *λpir*	Donnenberg and Kaper, 1991
E. coli DH5α λpir / pDS132	Host for cloning vector pDS132; *sup* E44, Δ*lacU*169 (Φ80*lacZ*ΔM15), *recA1, endA1, hsdR17, thi-1, gyrA96, relA1, λpir,* Cam^R	Simon et al., 1983
E. coli SM10 λpir / pDS132::Δ*rpfF*	Donor for transconjugation, harbouring construct pDS132::Δ*rpfF*, Cam^R	This study
E. coli SM10 λpir / pDS132::Δ*rpfR*	Donor for transconjugation, harbouring construct pDS132::Δ*rpfR*, Cam^R	This study

Plasmids		
pDS132	Low copy cloning vector<br>R6K *ori*, *mobRP4*, *cat*, *sacB*, Cam^{R}	Philippe et al., 2004
pDS132::Δ*rpfF*	Δ*rpfF* cloned into pDS132, Cam^{R}	This study
pDS132::Δ*rpfR*	Δ*rpfR* cloned into pDS132, Cam^{R}	This study
Primers		
rpfFmut1modf	5`- ACC TCT AGA CAC GAC ACC<br>ACT TCC GTG GCG - 3`	This study
rpfFmut2r	5`- TGA CTC GAG CGT AAT GAG<br>CTG ATG GAA ATC ACC - 3`	This study
rpfFmut3f	5`-AGA CTC GAG AGT CTT TCC TCA<br>TGT AAG TTA ACG - 3`	This study
rpfFmut5r	5`- TCG TCT AGA GGC GCT GCC<br>GTC GGT CCA GGC - 3`	This study
rpfFConf	5`- CGC CGC GTC CAC CCA GTC - 3`	This study
rpfFConr	5`- ATT AGT CTG ATC CTG CGC - 3`	This study
rpfRmut1f	5`- AGT TCT AGA CGC CTG ACG<br>CTG CTT ACG CAA ACC- 3`	This study
rpfRmut2r	5`- CCA CTC GAG GCG GCA GAC<br>CCG GTG CGG TCA CGG- 3`	This study
rpfRmut3f	5`- GAC CTC GAG GTG GAG AGT<br>GAA GAG GAA GAT GCG- 3`	This study
rpfRmut5r	5`- ACC TCT AGA GCC GGT TTC<br>ATC AGC GGC GAA ATC – 3`	This study
rpfRConf	5`- GAG AAT TAA GCG AAA TGG-3`	This study
rpfRConr	5`- GCG CTG CGT AAA GCA GGC-3	This study
Complementation		
C. turicensis LMG 23827^{T}	Template for amplification of *rpfF, rpfR*<br>CDS	Stephan et al., 2011
C. turicensis LMG 23827^{T}_<br>Δ*rpfF*	RpfF CDS mutant, cloning host for<br>pCCR9, pCCR9::*rpfF*	This study
C. turicensis LMG<br>23827^{T}_Δ*rpfF* / pCCR9	Mutant transformant harbouring low copy<br>cloning vector pCCR9, Tet^{R}	This study
C. turicensis LMG<br>23827^{T}_ Δ*rpfF* / pCCR9::*rpfF*	Mutant transformant harbouring construct<br>pCCR9::*rpfF*, Tet^{R}	This study
C. turicensis LMG 23827^{T}_<br>Δ*rpfR*	RpfR CDS mutant, cloning host for<br>pCCR9, pCCR9::*rpfR*	This study
C. turicensis LMG 23827^{T}_<br>Δ*rpfR* / pCCR9	Mutant transformant harbouring low copy<br>cloning vector pCCR9, Tet^{R}	This study
C. turicensis LMG<br>23827^{T}_Δ*rpfR* / pCCR9::*rpfR*	Mutant transformant harbouring construct<br>pCCR9::*rpfR*, Tet^{R}	This study
Plasmid		
pCCR9	Low copy cloning/expression vector, Tet^{R}	Randegger et al., 2000
Primers		
rpfFComplf	5`- TTT GGA TCC GCA AAG GGA<br>AAC AGA CGC- 3`	This study
rpfFComplr	5`- TTT AAG CTT GGT CAG CCA<br>GCT GCT GCG - 3`	This study
rpfRComplf	5`- GTA GGA TCC AAA CCC GCA<br>GCG TGA TCG- 3`	This study
rpfRComplr	5`- AAA TCT AGA CAG GCT AAC<br>GGC CAT GAC – 3`	This study
pCCR9-F	5`-TTT GAC AGC TTA TCA TCG-3`	Schwizer et al., 2013
pCCR9-R	5`-CCT ATG GAA GTT GAT CAG-3	Schwizer et al., 2013

Supplemental Table S2

Primers	Sequences	Source/Reference
Cturi_univ_16S_f	5`- GTG TTG TGA AAT GTT GGG T - 3`	Eshwar et al., 2015
Cturi_univ_16S_r	5`- ACT AGC GAT TCC GAC TT- 3`	Eshwar et al., 2015
csgA_f	5`- CGC AAT GGC ATT TCC T- 3`	This study
csgA_r	5`- GTT GTT GGC GAA ACC G - 3`	This study
bcsA_f	5`- AAA GGG CTC AAG CTC G - 3`	This study
bcsA_r	5`- TTG GAG TTG GTC AGG C - 3	This study
flhE_f	5`- GCG CTA TGA ACT GGC A-3`	This study
flhE_r	5`- GCG GTA GTT GAC GAT G-3`	This study

Supplemental Figure S1

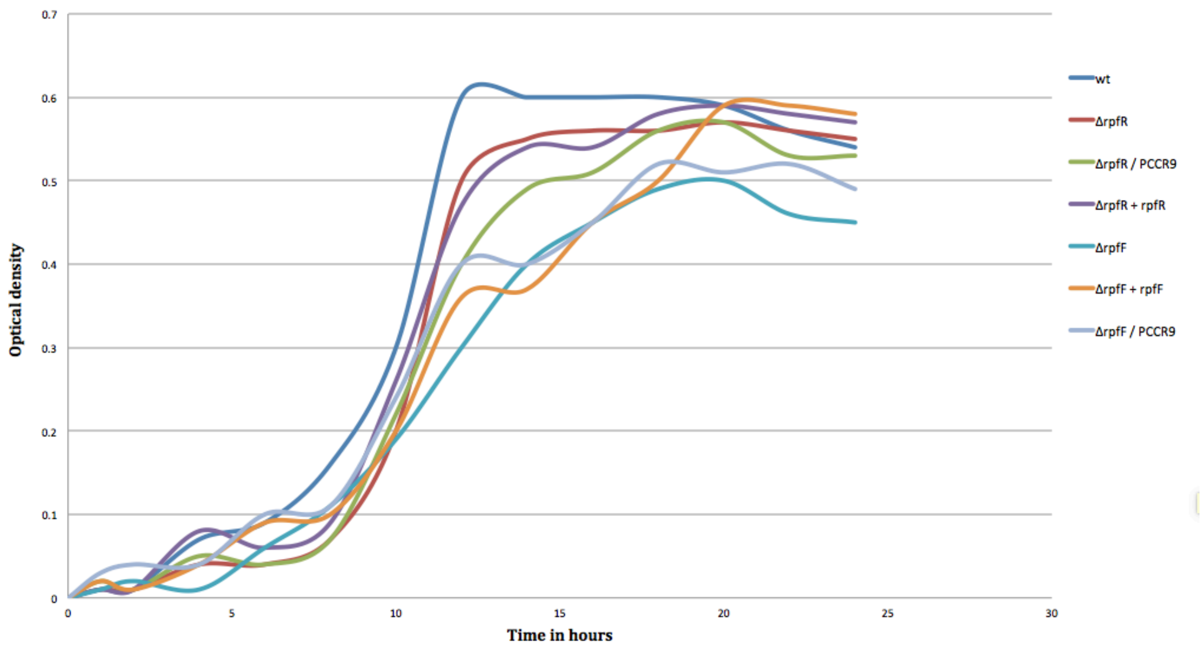

Table captions

Supplemental Table S1 Material used for mutant construction and complementation experiments.
Supplemental Table S2 Primers used for real time quantitative expression analysis.

Figure caption

Supplemental Figure S1 Growth curves of *C. turicensis* wt, mutants and complemented mutants as well as mutants carrying the pCCR9 vector only in AB minimal medium supplemented with 0.4% glucose and 0.5% casamino acids. Bacterial growth was monitored over 24 h at 30 °C and 600 nm in 200 µl volumes of medium supplemented in 96 well plates using the Bio-Tek microplate reader (Synergy HT; Bio-Tek, Germany).

References

1. Donnenberg, M. S. & Kaper, J. B. Construction of an *eae* deletion mutant of enteropathoenic *Escherichia coli* by using a positive selection suicide vector. *Infect. Immun.* **59,** 4310-4317 (1991).
2. Simon. R., Priefer, U. & Pülher, A. A broad host range mobilisation system for in vivo genetic engeneering: transposon mutagenesis in gram-negative bacteria. *Bio/Technology* **1**, 784-791 (1983).
3. Randegger, C. C., Keller, A., Irla, M., Wada, A. & Hächler, H. Contribution of natural amino acid substitutions in SHV extended-spectrum beta-lactamases to resistance against various betalactams. *Antimicrob. Agents Chemother.* **44**, 2759-2763 (2000).
4. Schwizer, S., Tasara, T., Zurfluh, K., Stephan, R. & Lehner A. Identification of genes involved in serum tolerance in the clinical strain *Cronobacter sakazakii* ES5. *BMC Microbiol.* **13,** 38 (2013).
